# Surface Inspection and Description in Metrology and Tribology—Vol.1

**DOI:** 10.3390/ma15165636

**Published:** 2022-08-16

**Authors:** Michal Wieczorowski, Maxence Bigerelle, Chris Brown, Pawel Pawlus, Rafal Reizer, Alejandro Pereira

**Affiliations:** 1 Division of Metrology and Measurement Systems, Faculty of Mechanical Engineering, Institute of Mechanical Technology, Poznan University of Technology, Piotrowo Street 3, 60-965 Poznan, Poland; 2 Laboratoire d’Automatique, de Mécanique et d’Informatique Industrielles et Humaines, LAMIH, Université Polytechnique Hauts-de-France, UMR CNRS 8201, 59300 Valenciennes, France; 3 Mechanical Engineering Department, Worcester Polytechnic Institute, Worcester, MA 01609, USA; 4 Faculty of Mechanical Engineering and Aeronautics, Rzeszow University of Technology, Powstancow Warszawy 8 Street, 35-959 Rzeszow, Poland; 5 Institute of Materials Engineering, College of Natural Sciences, University of Rzeszow, Pigonia Street 1, 35-310 Rzeszow, Poland; 6 Manufacturing Engineering Group (GEF) EEI Campus Lagoas, University of Vigo, 36310 Vigo, Spain

The primary goal of this Special Issue was to present recent trends in surface inspection and description, from both metrological and tribological points of view. Various research problems dedicated to surfaces in different scales were discussed. An important part of that were the techniques and methods that are and may be used for surface inspection and digitization (including free-form surfaces) [1]. These aspects are particularly important for Industry 4.0 and its purely metrological part, Metrology 4.0 with all aspects and consequences [2]. Another important topic was the separation of different frequencies, i.e., different ideas of filtration and multiscale approach to analysis [3,4]. This is particularly important for different ways of machining [5] and the investigation of bodies in contact [6,7], where tribological behavior and chemical processes on the surface are to be considered [8,9].

Every material object found in the human environment is bounded by surfaces [10]. Each such surface, in turn, is characterized by certain irregularities that occur on it and are an inherent feature of anything we can touch, know, or observe [11]. Some of them are visible to the naked eye, while others require the use of very sophisticated measuring instruments to determine their presence [12]. Irregularities can vary in nature, resulting from natural or human forces that seek to reduce it in some situations and the opposite in others, in order to achieve the desired effect. Surfaces are important in many different fields of science [13], often far beyond classical engineering topics from metal cutting, including emissive properties [14], wire arc hybrid manufacturing [15], concrete [16], bioengineering [17], or even archaeology [18,19].

Several decades ago, it was thought that, in a physical sense, surface topography or roughness was a systemically secondary problem and it was assumed that in engineering problems it would never play not only a primary role, but even an important one. Over time, the reality turned out to be different. This happened for two reasons. First, although it is a secondary issue, it encompasses a wide range of activities. Secondly, the issues called primary have already been solved, and it is increasingly apparent that the world we live in is dominated by secondary issues. Explaining this approach with an example, we can say that at present it is not a problem to build an internal combustion engine (a primary issue). The problem only begins when we want it to run for hundreds of thousands of kilometers, consume as little fuel as possible and emit a minimal amount of pollution into the atmosphere (a secondary issue). Surface irregularities are one of the factors in choosing the right manufacturing process, and, increasingly, manufactured components have surfaces with geometric structures made for specific applications. In such cases, the analysis at micro- or nano-scale is at least as important as the analysis of geometric dimensions [20].

The topography of machined surfaces has a decisive impact on the basic tribological properties of the surface, as well as tightness, fatigue strength, thermal and electrical conductivity, friction, deformation, joint stiffness, and positional accuracy, but also aesthetic characteristics (appearance). Using a metaphor from the borderline of poetry and forensics, it can be said that the arrangement of surface irregularities, is like a fingerprint of the manufacturing process [21].

Two types of methods are used to evaluate surface irregularities: profile and surface [22]. The former evaluate topography on the basis of a profile (series of profiles) or a sequence of images, and the latter on the basis of averaging and adopting a model describing the measured irregularities. The most widespread instruments for measuring unevenness of the geometric structure of a surface are currently contact profilometers, known for nearly a hundred years now [23]. Their place in industrial practice is firmly established by years of experience and a variety of applications and standardization procedures, which, until recently, were associated only with this particular measurement technique. In industry, the measurement technology used is often not taken seriously until it becomes a method that is recognized by suppliers and customers, will give repeatable, reproducible, and comparable results. In addition, the method should be adapted to the capabilities of the production laboratory or production conditions, and in this it should be possible to use even for personnel who are not always highly skilled. In research work, on the other hand, the trend of the last several years has been a gradual shift away from contact profilometry to the most diverse optical methods and microscopy based on atomic properties. The reason for this is, in the first place, the speed of measurement, which is much faster than is the case with contact methods. In addition, in a research setting we are dealing with people who, in the name of science, are willing to devote much more attention to the problem of measurement fidelity and reliability than instrument operators in industry. This behavior, of course, creates the possibility for many non-contact methods to slowly but consistently encroach on classical measurements for production purposes, and the coming years should confirm this. This is already becoming apparent in standardization work, where successive draft standards for topography measurements successively include new non-contact methods for surface roughness analysis.

The surface of any object being measured has features at the macro-, meso- and micro-nano scales. The Special Issue—Surface Inspection and Description in Metrology and Tribology—included articles on each of these, which shows, on the one hand, how wide the scope of the research being conducted is, and, on the other hand, how interconnected the different scales are and how they determine the functional behavior of surfaces and whole objects [24]. Starting from the micro-scale, Pawlus et al. [25] in their review regarding surface topography parameters and functions, showed that areal 3D analysis of surface texture gives more opportunities than a study of 2D profiles, and besides surface topography plays an important role in many fields of science and life. The evaluation of surfaces requires the use of different parameters and functions that can provide important information regarding functional behavior of surfaces in different applications [26]. The paper presents relationships among various surface parameters and functional properties. A proposal of a selection of parameters on the basis of their functional significations was also provided.

Moving towards the meso-scale, where the difference between surface irregularities and is less and less evident [27], we come to form errors, among which circularity has a prominent place. It is usually filtered out when measuring surface irregularities, but its nature often determines the functional aspects of cylindrical components. The various techniques for measuring roundness deviation include contact and non-contact techniques, among which air gauges are important. This is the subject of a publication [28]. Jermak et al. present in it the results of an investigation on the application of air gauges in the measurement of out-of-roundness parameters are presented. The principle of the measuring system is explained, and, in particular, the novel design of the floating gauge head is described. Considering possible sources of errors, simulations have been carried out, which helped to evaluate the influence of some of the parameters on the final measurement results. The accuracy parameters obtained showed highly satisfactory effects, considering short measurement time and non-contact method.

Surfaces were also important in the work on DPP 3D-Printed Injection Die for Investment Casting [29]. The investment casting method supported with 3D-printing technology, allows the production of unit castings or prototypes with properties most similar to those of final products. Due to the complexity of the process, it is very important to control the dimensions in the initial stages of the process. Various non-contact measurement techniques were used for the measurements (X-ray CT [30], structured blue-light scanner, and focus variation microscope) to avoid any additional damages to the injection die that may arise during the measurement. Dimensional accuracy analysis, form and position deviations, defect detection, and comparison with a CAD model were carried out. It is worth mentioning that X-ray computed tomography is more and more often considered as a very interesting method of surface analysis also in micro-scale [31,32]. Macro-scale was also analyzed in the paper prepared by Rekas et al. [33]. Here, a method for checking the geometry of stamped car body parts using a 3D optical measurement system was presented. The analysis focused on the first forming operation due to the deformation and material flow associated with stall thresholds. The geometry of car body elements was analyzed using an optical laser scanner. The control process allowed to correctly position the tools (punch and die), introducing the correction of technological parameters. This, in turn, has a fundamental influence on the specific features of the final product. The actual gap occurring between the forming surfaces based on the die and punch geometry used in the first stamping operation was also determined.

The study of geometric features related to the surface and feedback from manufacturing and tools also occurs in machining, since, in addition to geometry, the method of manufacturing is also important in describing surfaces. One new milling method that allows high feed rates and increased machining efficiency is high feed milling [34]. The method utilizes face cutters with a very small entering angle (10°–20°). Thus, the cut layer cross-section is different than in traditional milling. The study focused on analyzing the vibration amplitude, cutting force components in the workpiece coordinate system, and surface roughness. The experimental tests proved that, when milling with constant cut layer thickness, the high feed cutter allowed to obtain twice the cutting volume in comparison with the conventional face cutter. Krawczyk et al. [35] performed tests concerning turning on shafts made of Inconel 718. The tests were performed in dry and wet conditions, with changing cutting speed and constant feed and depth of cut. The shafts had different shapes: cylindrical, taper 30°, taper 45°, and sphere. The smallest roughness values (Ra and Rz) were obtained for larger angle between the surface and cutting edge. Cutting speed, machining conditions (dry and wet machining), as well as variable angle between the machined surface and the cutting edge influenced on the surface roughness: higher cutting speed and wet machining resulted in lower values of roughness parameters.

Grinding is another way of machining, which is used to obtain proper surface topography [36]. Lipinski et al. [37] presented an effectiveness analysis of the grinding process with the use of a new multi-layer abrasive tool. The tool consists of external layers with conventional structure aimed to decrease the grinding wheel load and ensure high volumetric efficiency of grinding. On the other hand, the task of the inner layer (containing 30% more abrasive aggregates) is to provide lower topography of the machined surface. Samples made of Ti-6Al-4V alloys were ground with the use of a multi-layer wheel and a conventional one. The set of parameters made it possible to differentiate surfaces obtained with the two different tools.

The topics of this Special Issue also dealt with issues related to tribology. One of the primary ones for which surface analysis plays a particularly important role is investigation of wear [38]. This was reported by many scientists for cutting tools [39], or working pairs [40], including numerical and qualitative characterization [41]. In this book, Hawryluk et al. [42] presented an analysis of the durability of punches applied when manufacturing chromium–nickel steel valves for combustion engines by forging in two operations—coextrusion of a long shank, and forging valve head in closed dies. Due to intensive abrasive wear, as well as high adhesion of the forging material to the tool surface durability of the forging tools is rather low. Among other techniques of investigation, the Authors performed 3D scanning of tool sections, to extend the operation life of forging tools. Wear was also analyzed by Tecza [43] for Hadfield cast steel. This material is characterized by high wear resistance, when subjected to the effect of dynamic loads. On the other hand, during unloaded abrasion, its wear resistance is very low, comparable to carbon cast steel. To increase the wear resistance the Author used primary vanadium carbides to obtain a two-phase structure after solidification. These carbides, evenly distributed in an austenitic or austenitic–martensitic matrix, significantly increase the wear resistance, even in very harsh conditions.

Another tribology application was investigated by Czapczyk et al. [44]. The Authors presented the results of mechanical and tribological tests of Ni-P/Si_3_N_4_ nanocomposite coatings, that were deposited on the AW-7075 aluminum alloy. Abrasive wear was tested and determined in the reciprocating motion using the “ball-on-flat” method, while surface topography was examined by means of a profilometer. In the research it was found that the Ni-P/Si_3_N_4_ layers produced in the bath with the Si_3_N_4_ nanoparticle content in the amount of 2 g/dm^3^ are more resistant to wear and show greater adhesion than the Ni-P/Si_3_N_4_ layers deposited in the bath with 5 g/dm^3^ of the dispersion phase. The same AW-7075 aluminum alloy was one of the materials examined by Pereira et al. [45], who tried to replace aluminum welding operations with adhesive operations of other types of materials, such as polyamides (TEPEX^®^—Dynalite 202-c200/50% TYP 13, Composite manufactured by LANXESS Deutschland GmbH from Koln, Germany). The Authors concentrated on texturing of substrate made in 7075 aluminum specimens for structural adhesion with TEPEX by AF-163-2 film. The tests were based on the topography measurement of the surfaces to be joined, and the uniaxial shear tests of adhesive samples. The results confirmed a significant correlation between the texture parameters of initial surfaces and maximum shear stress.

Tribological behavior is also important for biomedical applications. Schmeidl et al. [46] performed study aimed to determine the kinetic frictional force of the TiNbTaZrO (Gummetal) orthodontic wire and compare it to the wires made of stainless steel, nickel–titanium, cobalt–chromium, as well as titanium–molybdenum alloys. The dynamic frictional forces between the brackets and ligated wires were measured utilizing a specialized tensile tester machine. Surface topography of the wires were inspected by means of a focus variation microscope. The Authors found out that bendable TiNbTaZrO wire may be used for sliding mechanics due to its favorable frictional properties and good topographical characteristics.

Even from this brief description, it is clear how important it is to properly approach surface topography at different scales and understand its functional aspects. The surface, moreover, is increasingly considered not as a rigid boundary between objects or an object and its surrounding environment, but as a soft interface, especially considering the atomic or molecular scale. This is particularly evident with non-contact measurement methods using electromagnetic waves (in or out of the visible range). Then, considering that light has a dual nature of corpuscular-wave, and that its molecules touch the surface interacting with it raises the philosophical question of how contactless these methods are. This creates one of many avenues for the development of methods for surface measurement and analysis, showing that the future in this field is wide open.

## Data Availability

Data sharing is not applicable to this article.
